# Spring cleaning as a safety risk: results of a population-based study in two consecutive years

**DOI:** 10.1186/1471-2458-11-631

**Published:** 2011-08-06

**Authors:** Soheil Saadat, Mojgan Karbakhsh

**Affiliations:** 1 Sina Trauma Research Center, Tehran University of Medical Sciences, Tehran, Iran; 2 Department of Community Medicine, Tehran University of Medical Sciences, Tehran, Iran

**Keywords:** spring cleaning, injuries, home, houseclean, Iran, prevention

## Abstract

**Background:**

Spring cleaning is a popular tradition in Iran as well as in many other countries. The purpose of our study was to determine the pattern and compare the incidence of spring cleaning related injuries in Tehran, in the years 2007 and 2008.

**Methods:**

In the year 2007, a household survey was performed in Tehran by random cluster sampling. The survey was repeated in May 2008 with the same clusters and starting points, but different households. The incidence of spring cleaning related injuries, the age and sex of injured person(s), the mechanism, type and cost of injuries were recorded through semi-structured interviews. The incidence rates of injuries and injuries leading to health visits (severe) according to sex and age groups were calculated. Data were analyzed using SPSS and STATA statistical softwares.

**Results:**

The incidence of all and severe spring cleaning related injuries were 3.8 (3.0 - 4.8) and 1.6 (1.1-2.3) per 1000, respectively. The most common mechanisms of injuries were falls, followed by cutting and lifting heavy objects or overexertion. Falls were also the main mechanism of severe injuries. The most common injuries were open wounds, followed by superficial injuries (including contusions) and sprain and strain. Among severe injuries, the most frequent injuries were open wounds and contusions, followed by dislocations. The injuries were most common among women with an incidence of about 8.4 per 1000 in women older than 18 years of age (severe injuries: 3.4 per 1000 (2.2-5.1)).

**Conclusion:**

The incidence of spring cleaning related injuries is high enough to raise concern in health system authorities. It could be estimated that about 23,927 to 38,283 persons get injured during the spring cleaning in Tehran at the beginning of every Persian new year. In addition, about 8,773-18,344 of these cases are expected to be severe enough to lead to medical attention (considering 7,975,679 as the population of Tehran at the time of study). Improving awareness of families, especially young women, regarding the scope and importance of spring cleaning safety can be suggested as the first population-based strategy to decrease the incidence of these injuries.

## Background

Spring cleaning is a popular tradition widely practiced by many nations throughout the world such as Northern Europeans and Americans. In Iran, It is also considered a nationwide tradition to which nearly no families are exceptions. The Persian's new year starts with spring and Iranians spend the last few weeks of the year (in winter) cleaning their houses and surroundings to make them ready for a new year. "Khaané Tekaani" is the Persian term used for spring cleaning which literally means "shaking the house".

A good spring cleaning often means climbing, lifting heavy objects and using strong cleaning solutions [1] which can all potentially lead to serious injuries. This means that a large proportion of population is exposed to the risk of injury in a rather short period of time. Previous studies have shown that home can be an important setting for unintentional injuries especially for children, women, and elderly who spend a considerable time at home [2-6]. Nevertheless, there are no published papers regarding the scope and burden of spring cleaning injuries. As these injuries occur at home, their recognition highlights the importance of safety at home. In addition, there seems to be little understanding on behalf of the general public about the potentially high risks that they are exposed to during spring cleaning and this adds to the importance of spring cleaning as a neglected health issue. For instance, a survey performed by the national nonprofit Home Safety Council in the United States showed that only six percent of adults recognized the increased risk for home injuries during the spring as compared with other seasons, leaving the vast majority of people in danger [7].

Although home injuries are reported with various rates from different parts of the world, they are rather prevalent. For instance, a Pan-European housing and health survey undertaken from 2002 to 2003 in eight European cities revealed that about 25% of all surveyed residents had a home accident during the year prior to the survey [8]. In Turkey, a population-based survey estimated the annual incidence of unintentional non-fatal home-related injuries at about 10.8% [9]. During 2006 in Korea, home injuries comprised 21.1% of total daily life injuries [10]. In 1998 in the United States, there were more than 12 million unintentional home injuries requiring some form of medical attention with an incidence rate of 4,804 per 100,000 [11]. In the same year, the annual incidence rate of healthcare-reported home-related injuries in urban areas of Iran was about 390 per 100,000 and the fatality rate was 2.8 per 100,000 [12].

The purpose of our study was to determine the pattern and compare the incidences of spring cleaning related injuries in Tehran, in the years 2007 and 2008. Our study aimed to answer the following questions:

"What is the burden of spring cleaning related injuries in the studied population, regarding the mortality, morbidity and costs?", "who are the main high risk groups for being injured in spring cleaning?", "what types and mechanisms of injuries are more common?" and "what proportion of the studied population had received related preventive advice?".

## Methods

A household surveys was arranged eight weeks after the beginning of Persian new year in May 2007 by random cluster sampling. The sampling frame for the study was the postal address registry of metropolitan city of Tehran, Iran. One hundred addresses were selected randomly and each address served as the starting point of the survey in every cluster. The interviewers moved counterclockwise from the starting address to capture 25 houses in each cluster. To increase the diversity within each cluster and reduce the design effect, the interviewers skipped 3 houses and included the fourth house in the study.

The survey was repeated in May 2008. This time, the same clusters and starting points were used; but, different households were included as the interviewers moved clockwise from the starting address. Other settings were identical in both years. The inclusion criteria were presence of at least one parent at home at the time of interview and agreement for participation in the study. If there were no parents at home or they refused to participate in the study, then that house was skipped.

Data was collected using a semi-structured interview. The respondents were asked if in their household, they had practiced spring cleaning at the beginning of the recent new Persian year and if any related injuries or deaths had occurred. If the answer to this latter question was "yes", they were asked about the age and sex of injured person(s), the mechanism, type and cost of injuries and how the injury was managed. The injury was considered "severe" if it had led to a visit by a health care practitioner. In addition, the respondents were asked if they had ever received any information on safe spring cleaning and if so, what had been the sources of information. The regional price of housing in the residential area of study subjects was gathered from the Iranian ministry of housing and urbanization. The regional price of housing was used as a determinant of socioeconomic situation of the household. Population of Tehran at the time of study was considered to be about 7,975,679 for estimating the overall number of injured cases in Tehran.

Injuries and mechanisms were coded according to the International Classification of Diseases and related health problems, tenth version (ICD-10) by the main investigator. The incidence rates of injuries according to sex and age groups were calculated. Incidence rates were also provided for ages 18 and above. This categorization of age was used as persons under the age of 18 are seldom directly involved in spring cleaning activities in Iran and overall estimations might lead to dilution of association. Nevertheless, no age groups (e.g. children) were excluded; as it is not rare that they become unintentionally exposed to injuries during spring cleaning.

Data were analyzed using SPSS statistical software (version 13). Confidence intervals were calculated assuming Poisson distribution using STATA 8 SE software.

### Ethical Approval

The proposal of this research was approved by the research committee of Sina Trauma Research Center affiliated to Tehran University of Medical Sciences. Each potential subject was adequately informed of the aims, methods, sources of funding, institutional affiliations of the researchers and the anticipated benefits of this research for the society. He/She was also informed of the right to refuse to participate in the study. The interview was performed after ensuring that the potential participant has understood the information and is willing to participate.

## Results

The survey included 4920 households consisting of 18275 individuals (9100 included in 2007 survey and 9175 included in 2008). Overall, 93.6% (92.9 - 94.3) of households reported involvement in spring cleaning at the beginning of the preceding Persian new year (93.2% in 2007 and 94.0% in 2008). In 14% (12.7-15.4) of households, father of the family had been responsible for performing spring cleaning, while in 36% (34.1-37.9) only mother of the family and in 42% (40.1-44.0) mainly the mother with the help of other family members had done spring cleaning. In 8 percent (6.9-9.1), non-professional workers had been hired for spring cleaning.

Fortunately, no deaths which could be attributed to spring cleaning were reported in the selected households. The incidence of all and severe spring cleaning related injuries were 3.8 (3.0 - 4.8) and 1.6 (1.1-2.3) per 1000 population, respectively. This incidence of all and severe injuries were 4.1 and 1.3 per 1000 in the year 2007 and 3.5 and 2.0 per 1000 in 2008. There were no significant differences in spring cleaning practice and spring cleaning injury incidence between the years 2007 and 2008 (P > 0.05).

Overall, 70 people had been injured in spring cleaning in the two consecutive years, 38 cases in 2007 and 32 in 2008. Twelve injured persons in the year 2007 and 18 in the year 2008 were referred to medical facilities for further treatment. Fifty three of injured persons (75.7%) were mothers and seven (10.0%) were daughters of the family. The mean age of female injured persons was 35.4 ± 12.1 and that of males was 24.5 ± 13.9 years (P < 0.05).

Only 4.4% of households had received education/instructions on safe spring cleaning. The sources of safety information were mentioned as yellow (non-educational) magazines (2.2%), TV/Radio programs (1.3%), former education in school (1.1%) and an expert family member (0.4%).

The most common mechanisms of injuries were falls, followed by cutting and lifting heavy objects or overexertion. Falls were also the main mechanism of severe injuries (40.0%) (Table 1). The most common injuries were open wounds, followed by superficial injuries (including contusions) and sprain and strain. Among severe injuries, the most frequent injuries were open wounds and contusions (46.6%), followed by dislocations (13.3%).

**Table 1:** Distribution of spring cleaning related injuries (listed according to ICD code) by study characteristics

*Mechanism*	*n*	*Mean age*	*Female/male*	*Number needing medical attention*
Fall on same level	3	33.5	3/0	1
Fall involving chair	10	28.2	4/1	6
Fall involving other furniture	4	44.0	3/1	3
Fall from stairs	3	52.5	3/0	2
Struck by falling object	5	30.7	5/0	3
Crushed between objects	7	32.0	6/1	2
Contact with sharp glass	8	34.3	3/1	1
Contact with non-powered hand tool	5	34.7	4/1	3
Contact with other powered hand tools and household machinery	2	25.5	2/0	2
Contact with hot tap-water	5	39.3	5/0	2
Overexertion and strenuous or repetitive movements	15	28.9	13/2	3
Exposure to other specified factors	3	60.0	2/1	2

**Total**	**70**	**33.8**	**6/1**	**30**

There was one case of tracheal irritation due to inhalation of whitener liquids vapor and another case of chemical corrosion of hands with hydrochloric acid which was used as whitener. In addition, a case of ocular injury had happened due to fall of a heavy object.

There were three cases with fractures and four cases with dislocation. All fractures had occurred in women aged 18, 43 and 70 years and had been due to fall from ladder, chair and from the same level (following slipping), respectively. The affected anatomical regions in fractures were neck of femur, pelvis, and ankle. Three dislocation cases were females, all injured due to fall.

The incidence rates of injuries according to gender and age are displayed in Table 2.

**Table 2:** Incidence rate of spring cleaning related injuries in the years 2007 & 2008, Tehran

*Age-Sex group*	*Population in the sample*	*Spring cleaning injuries*		*Incidence rate, per 1000 (95% CI)*		*Tehran population*
		***All***	***Severe***	***All injuries***	***Severe injuries***	

All male	9,400	10	5	1.1 (0.5 - 2.0)	0.5 (0.2-1.2)	4,077,719
All Female	8,875	60	25	6.8 (0.5 - 8.7)	2.8 (1.8-4.2)	3,897,960
Male ≥ 18	7,446	8	5	1.1 (0.5 - 2.1)	0.7 (0.2-1.6)	3,083,404
Female ≥ 18	7,059	59	24	8.4 (6.6 - 10.8)	3.4 (2.2-5.1)	2,954,250

**Total**	**18,275**	**70**	**30**	**3.8 (3.0 - 4.8)**	**1.6 (1.1-2.3)**	**7,975,679**

Injuries occurring in males consisted of open wound of fingers (n = 2) and thigh (n = 2), dislocation of ankle due to fall (n = 1), contusion of shoulder and upper arm (n = 2), sprain and strain of lumbar spine due to lifting heavy objects (n = 2) and sprain/strain of other and unspecified parts of lumbar spine and pelvis due to overexertion (n = 1). Two of male injured persons were the father and eight others were sons of the family.

The mean out-pocket medical expenses of injuries were 37.7 US dollars (SD: 123.5, median: 5.4) (1 US dollar was equal to 9,250 Rials (the Iranian currency) at the time of the study). People who reported a fall injury had spent 55.9 (SD: 164.9) US dollars out of pocket on medical care compared to 16.2 (SD: 31.9) US dollars in those who had injured due to other mechanisms; but the difference was not statistically significant (P >0.05; Power: 0.177).

The mean housing price in residential area of households that reported a spring cleaning related injury was 1749.9 ± 630.9 USD per square meter, compared to other households which was 2162.7 ± 965.6 USD per square meter (P < 0.01).

## Discussion

This is among the first reports on the incidence and pattern of spring cleaning injuries. It can be estimated that each year about 23,927 to 38,283 persons get injured during spring cleaning in Tehran of whom about 8,773-18,344 seek medical attention. The observed incidence of spring cleaning related injuries (3.8 in 1000) is high enough to raise concern in health system authorities. Although the exposure time of community is fairly short, this incidence has been even higher than the rate of road traffic injuries in 2007 (343.1 in 100,000) [13].

Annual rates of home injuries reported from different countries throughout the world vary from 1.5% in 25-64 year old inhabitants of Stavanger, Norway to 10.8% In Turkey [9,11,14-17]. Comparison of these estimates with the incidence of spring cleaning injuries that arise within the period of a week or two highlights the importance of spring cleaning as an important contributor to home injuries.

There are some possible explanations for this observed high incidence rate. Firstly, a large number of people get involved with spring cleaning and are exposed to its consequences. Moreover, people may not perceive house cleaning as a high risk activity and most of them do not care about safety measures or hiring trained workers. This has also contributed to the preponderance of falls as the most common mechanism of injuries in our setting, similar to some reports on home injuries [6,8-10].

Spring cleaning related injuries were more frequent among young women with an incidence of about 8.4 per 1000 in women older than 18 years of age (3.4 per 1000 for severe injuries). Women have already been recognized as the high risk group for home-related injuries [9,10,12,14,15,18]. Regarding the high risk age groups, home injuries are generally seen among the youngest and oldest subjects [9,14-17], while cases with spring cleaning injuries were in their 20s and 30s. The mean age of injured males was significantly lower than females. This might be due to the key role of females -as mothers- in spring cleaning and males assisting them as household members or young manual workers hired for help.

The economical toll of spring cleaning related injuries seems considerable. The mean out of pocket medical expenses of these injuries were 37.7 US dollars (with Iran's per capita GNI estimated at 4120 US Dollars in 2008 [19]). In Iran, people have to pay at least fifty percent of their health expenditures out of pocket [20]. Thus, the total costs might be about two times of that reported by study subjects. Direct and indirect costs of home injuries are also high in other countries [14,21]. In New Zealand, unintentional home injuries impose an annual social cost of about 3.5 times the annual social cost of road injuries [22]. This economic burden does not seem to be equally shared by different income levels. In our study, similar to two reports on home injuries from Turkey, participants with low socioeconomic status were among the high risk groups for being injured [9,23]. Home injuries due to falls impose higher costs than other home injuries according to some reports [21]. This did not prove to be significant in our research due to lack of power for this hypothesis.

Our study had some limitations. It was mainly based on self reports of families regarding any injuries occurring on the preceding spring cleaning which was two months prior to the survey. Nevertheless, significant injuries are not easily forgotten and are less likely to lead to recall bias. Moreover, our attempt to show the incidence of injuries in two consecutive years can add to the reliability of findings. Another potential shortcoming might be that our study did not distinguish between occupational injuries (among workers hired to take care of house cleaning) and non-occupational injuries. As in our setting, the prevalence of having workers at home for spring cleaning was only about 8 percent, this limitation is not expected to be of great importance in interpretation of results. Further research aiming to distinguish patterns and attributes of occupational and non-occupational spring cleaning related injuries can be suggested.

Spring cleaning-related injuries are preventable in most cases but seem to be neglected by health authorities as well as the public, maybe because the communities get exposed to these injuries just once in a year. Although more than 93 percent of households had done spring cleaning, only a minority had received education on safe spring cleaning. In some countries e.g. in the US, people have access to information regarding safety measures for spring cleaning according to the pattern and burden of these injuries [24,25]. Improving awareness of families especially housewives regarding the scope and importance of spring cleaning can be suggested as the first population-based strategy to decrease the incidence of these injuries. Use of safe equipment and materials should be promoted. Moreover, safety standards of these equipments and materials should be stressed upon and inspected by municipality authorities.

## Conclusion

Spring cleaning is a safety risk especially for young women and seems to be neglected. Its incidence is considerably high in Iran as a result of involvement of family members and non-professional people in spring cleaning in a short period of time. The incidence of these injuries is higher than reputed injuries i.e. transport related injuries and deserves special attention. Improving awareness of families and health authorities followed by promotion of safe and standard equipment and materials should be considered.

## Conflicts of interests

The authors declare that they have no competing interests.

## Authors' contributions

The main idea belonged to the first author (SS). He also carried out the design of the study, coordinated and conducted the study and participated in preparation of the manuscript. MK as the second author participated in the conduct of the study and participated in preparation of the manuscript. Both authors read and approved the final manuscript.

## Pre-publication history

The pre-publication history for this paper can be accessed here:

http://www.biomedcentral.com/1471-2458/11/631/prepub
